# Functional Associations and Resilience in Microbial Communities

**DOI:** 10.3390/microorganisms8060951

**Published:** 2020-06-24

**Authors:** Maria-Luisa Avila-Jimenez, Gavin Burns, Zhili He, Jizhong Zhou, Andrew Hodson, Jose-Luis Avila-Jimenez, David Pearce

**Affiliations:** 1 Biology Department, University Centre in Svalbard, 9171 Longyearbyen, Norway; mlavilaj@gmail.com; 2 British Antarctic Survey, Cambridge CB3 0ET, UK; gavinburns@gmail.com; 3 Department of Microbiology and Plant Science, Institute for Environmental Genomics, University of Oklahoma, Norman, OK 73019, USA; hezhili@mail.sysu.edu.cn (Z.H.); jzhou@ou.edu (J.Z.); 4 Environmental Microbiomics Research Center and School of Environmental Science and Engineering, Southern Marine Science and Engineering Guangdong Laboratory (Zhuhai), Sun Yat-Sen University, Guangzhou 510006, China; 5 Geology Department, University Centre in Svalbard, 9171 Longyearbyen, Norway; Andrew.Hodson@UNIS.no; 6 Department of Environmental Sciences, Western Norway University of Applied Sciences, Røyrgata 6, N-6856 Sogndal, Norway; 7 Department of Informatics and Numerical Analysis, University of Cordoba, Campus Universitario de Rabanales, Carretera Nacional IV, Km. 396. C.P. 14014 Cordoba, Spain; jlavilaj@gmail.com; 8 Department of Applied Sciences, Faculty of Health and Life Sciences, Northumbria University at Newcastle (UK), Ellison Building, Northumberland Road, Newcastle-upon-Tyne NE1 8ST, UK

**Keywords:** resilience, functional diversity, redundancy, Antarctic bacteria, stability

## Abstract

Microbial communities have inherently high levels of metabolic flexibility and functional redundancy, yet the structure of microbial communities can change rapidly with environmental perturbation. To understand whether such changes observed at the taxonomic level translate into differences at the functional level, we analyzed the structure of taxonomic and functional gene distribution across Arctic and Antarctic locations. Taxonomic diversity (in terms of alpha diversity and species richness) differed significantly with location. However, we found that functional genes distributed evenly across bacterial networks and that this functional distribution was also even across different geographic locations. For example, on average 15% of the functional genes were related to carbon cycling across all bacterial networks, slightly over 21% of the genes were stress-related and only 0.5% of the genes were linked to carbon degradation functions. In such a distribution, each bacterial network includes all of the functional groups distributed following the same proportions. However, the total number of functional genes that is included in each bacterial network differs, with some clusters including many more genes than others. We found that the proportion of times a specific gene must occur to be linked to a specific cluster is 8%, meaning the relationship between the total number of genes in the cluster and the number of genes per function follows a linear pattern: smaller clusters require a gene to appear less frequently to get fixed within the cluster, while larger clusters require higher gene frequencies. We suggest that this mechanism of functional association between equally rare or equally abundant genes could have implications for ecological resilience, as non-dominant genes also associate in fully functioning ecological networks, potentially suggesting that there are always pre-existing functional networks available to exploit new ecological niches (where they can become dominant) as they emerge; for example, in the case of rapid or sudden environmental change. Furthermore, this pattern did not correlate with taxonomic distribution, suggesting that bacteria associate based on functionality and this is independent of its taxonomic position. Our analyses based on ecological networks also showed no clear evidence of recent environmental impact on polar marine microbial communities at the functional level, unless all communities analyzed have changed exactly in the same direction and intensity, which is unlikely given we are comparing areas changing at different rates.

## 1. Introduction

Functional diversity includes all biological processes performed within an ecosystem and it has been suggested that the more functionally diverse an ecosystem is, the more resilient it is to environmental change [1]. However, functional diversity studies based on ecological networks have suggested that most ecosystems include a relatively small core set of functions (whose depletion could lead to ecological collapse), and a large set of peripheral functions (that can be depleted without an observable impact on the ecosystem)—such a pattern has been termed a scale-free network [2]. Following on from this, an increase in overall functional diversity should also lead to a proportional increase in these peripheral functions (whose depletion would not impact the core function of the ecosystem) hence conferring resilience. Such a mechanistic link has not been tested previously.

Microbial communities are ideal systems in which to test ecological theories, for their relative simplicity and the direct link between genetic composition and ecological function [3]. Understanding functional resilience and the potential impact of changes in microbial community structure and ecology remains a major challenge, as microbes tend to show high levels of both metabolic flexibility and functional redundancy [4]. Despite this, microbial community composition changes rapidly with environmental perturbation and they appear to show little tendency to return to the original structure following such a disturbance [5,6]. This could be due to changes in the competitive advantage in co-occurring microorganisms (e.g., [7]). Conversely, recent studies have also found unexpected levels of ecological resilience, such as within sub-Arctic phytoplankton communities [8] and the reason behind such conflicting results could partly be because traditionally microbial resilience tends to be measured in terms of their ability to recover back to an original state after a disturbance based on taxonomy, i.e., community composition [6] or population dynamics [9,10] alone. To complicate matters, functionality is also still commonly assessed using environmental conditions as proxy [11] or by overly relying on taxonomic markers [12,13,14]. In any case, the potential impact that changes at the microbial level could have on upper trophic levels would strongly depend on whether the remaining taxa are able to maintain the same level of ecological function (ecosystem service) as the original community.

Polar ecosystems represent an ideal platform to test such hypotheses about ecological resilience, as they are often considered fragile or close to ecological tipping points [15,16,17,18]. For example, functional redundancy is predicted to be low in Antarctic soils [17], while polar marine ecosystems are thought to be potentially close to ecological collapse as a consequence of rapid sea ice loss and temperature fluctuations [18,19]. Furthermore, each microbial species is predicted to fulfil distinct ecological functions in these comparatively species-poor environments [17,18], representing a system which, in theory, is particularly vulnerable to environmental change and biodiversity loss.

In this study, we analyzed the structure of functional groupings (as defined in [20,21] and summarized in Supplementary Table S1) arising from common methods to measure ecological resilience, such as network analysis and cluster analysis, to identify whether;

(1) there is a link between network structure and the distribution of functional genes;

(2) the level of functional redundancy in this system is indicative of either resilience or a close to ‘collapse’ scenario;

(3) there are any signs of deterioration in the investigated microbial system at the functional level.

Thus, we analyzed the network structure and distribution of microbial functions based on the co-occurrence of functional genes (as defined in [20]) across a broad geographical range. This analysis allowed the identification of specific characteristics of the functional network that could explain the microbial ability to survive and potentially thrive even in the most extreme environments on Earth.

## 2. Materials and Methods

### 2.1. Sampling

A quantity of 300 L of seawater was collected 30 m below the surface (at the chlorophyll maximum) using a CTD (Conductivity, Temperature, and Depth) sensor, from 10 high latitude locations (the Antarctic locations are shown in Figure 3; the Arctic location was at the water confluence between Kongsfjord and Krossfjord in the West Coast of Svalbard, Norway).

The cruise data were deposited and are available at the BODC (British Oceanographic data Centre), including cruise reports, sampling station locations and cruise tracks for the following relevant oceanographic cruises: JR144 (26/2/06–17/4/06) to the Scotia Sea and the South Sandwich Islands; IPY Kinnvika RV Horyzont II to Magdalenefjord, Krossfjord and Kongsfjord, Svalbard (9/7/08–13/7/08); JR179 (21/2/08–11/4/08) to the Amundsen Sea, Bellingshausen Sea and Pine Island Bay; JR262 (24/9/11–20/11/11) to South Georgia and the South Orkney Islands; and JR230 (02/12/09–11/12/09) to the Bellingshausen Sea and Adelaide Island. This represents a sampling acquisition effort of 6 cruises and 50 days of sampling covering areas above and below the Southern Antarctic Circumpolar Current and above the Arctic Front.

The sample water was passed through a sonication bath to disrupt suspended particles and through a 2.7 µm prefilter using a sterile stainless-steel housing. The water was then subjected to ultrafiltration in a Pellicon 2 ultrafiltration system (EMD Millipore, Billerica, MA, USA) with 1 × 10,000 Da molecular weight cut-off filters. The permeate was excluded and the retentate recycled through the system until the total seawater volume reached <300 mL, and frozen at −20 °C for further analysis. The 300 mL retentate was defrosted slowly and centrifuged at 38,000 revolutions per minute (r.p.m.) to recover whole cells in a centrifugal concentrator. The DNA from cells concentrated in this way was subjected to DNA extraction using the MoBio water DNA extraction kit (MoBio, Carlsbad, CA, USA) according to the manufacturer’s instructions.

### 2.2. Microarray

To characterize the functional composition of the microbial communities in the marine samples investigated, we used a functional gene array (GeoChip 3.0, [20]), a high throughput metagenomic tool to detect and characterize microbial communities. We chose this microarray for its high specificity, low false-positive rates, inherent signal quantification and to eliminate potential observer bias. The full design pipeline for the functional chip array is previously described in [20], whereby CommOligo 2.0 [22] was used to design both gene- and group-specific probes, which were subsequently synthesized by Invitrogen (Carlsbad, CA, USA) and arrayed onto Corning UltraGAPS (Corning, NY, USA) slides using a Microgrid II Arrayer.

GeoChip 3.0 targets a variety of functions involved in carbon, nitrogen, phosphorus and sulphur cycles, energy metabolism, antibiotic resistance, metal resistance and organic contaminant degradation [20], which are considered relevant to those selected ecosystems, especially for biogeochemical cycling genes. Therefore, this selection of functions also covers major environmental processes. Even if we could expect the polar oceans to hold a proportion of genes, microorganisms and pathways not present elsewhere, and consequently not detectible by the GeoChip, we can still conclude that this pattern of modular resilience holds for all the main biogeochemical processes identified for microbial communities worldwide. This also opens the question about whether the pattern would hold for community-specific genes and pathways. More interestingly, if future studies show that this pattern does not hold for community-specific functions, this could further show why this set of functions and not others have evolutionarily become biogeochemically important worldwide. As we used DNA samples instead of mRNA for GeoChip analyses, these data are interpreted as being the functional potential instead of functional activity, drawing in the pool of functional pre-adaptations available within the community studied for these essential functions. In addition, the level of accuracy of the GeoChip 3.0 is to be considered, as it can detect down to 1 or 2 cells [21].The genes in the microarray were selected to cover a large proportion of common functional genes involved in major known ecological and environmental processes across the world.

### 2.3. Taxonomic and Species Richness Analysis

As a taxonomic marker, we used the gene *gyrB* encoding DNA gyrase b-subunit as the phylogenetic marker on GeoChip 3.0. Sequences of 16S rRNA genes are widely used as phylogenetic markers for bacterial/archaeal systematics and ecology [23]; however, it is difficult to use them to obtain fine-scale resolution at species and/or strain level as is required here. A phylogenetic tree based on *gyrB* results in a magnitude higher resolution than a tree based on 16S rRNA genes [24,25]. *GyrB* is used here as an indicator of taxonomic diversity. This way the phylogenetic markers could be used to conduct a similar analysis to the functional genes and test whether the pattern for functional genes can be explained by taxonomical distribution alone.

The pattern of presence/absence of taxa based on this marker was used to assess whether the sites differed in relation to species distribution and richness. Thus, species richness index was estimated for each site. A pairwise *t*-test with Holm adjustment (significance level *p* < 0.05) was performed to assess the distribution of taxonomic species and individual functional genes across sites.

### 2.4. Network Construction and Cluster Analysis

We used weighted gene co-expression network analysis (WGCNA) and KMeans++ clustering to identify whether, at the functional level, microbial communities are arranged in distinguishable interactive units (modules or clusters). WGCNA is a widely used data mining method, especially for studying biological networks based on pairwise correlations between variables. Network analyses are particularly useful to represent the level of interconnectivity within interactive units. We used WGCNA as it was tailored specifically for microarray data [26].

The groups formed using WGCNA are known as modules, while the groups identified by KMeans++ are defined as clusters. In both cases, they will correspond to a set of bacteria, or a bacterial network, whose genes are highly likely to interact within the environment as they tend to co-occur.

We used the specifically designed WGCNA R package for network detection, gene selection and network construction [26,27,28]. A total of 4915 functional genes were included in the network analysis. These were the genes present in at least six of the sites, and hence useful to establish meaningful multiple correlations. After assuming an initial scale-free topology, we identified a soft threshold power of 10 as the highest power showing a significant increase in mean connectivity among module items. We used the module eigengenes to plot the network heatmap.

WGCNA allocates to a module all genes that tend to co-occur. If a specific gene is found more than a threshold number of times (80%) in the same sample than another gene, these two genes are linked together and grouped in the same module. Each gene included in the analysis also fulfils a specific function (i.e., antibiotic resistance, carbon sequestration, etc.) for being an essential part of a metabolic path related to the allocated function. Note that the GeoChip was specifically designed to include genes unambiguously assigned to a unique function.

To ensure that the pattern found was not an artefact of the clustering method used, we re-analyzed the data using a completely different clustering method, known as the K++ mean. K++, as distinct from WGCNA, can allocate a gene to more than one cluster instead of allocating the gene to the first group where the threshold is met. Therefore, the order in which the genes are included in the analysis has a lower influence in the result than for WGCNA.

Thus, for the second clustering analysis (results in Figure 3), the KMeans++ clustering method with seeding [29] was implemented in the data mining software WEKA [30]. WEKA compiles a collection of machine learning algorithms tailored for data mining, whilst the KMeans++ method applied implemented these algorithms to find the centroid for each cluster with iterations (repeated until the process provides stable results). The centroids are originally chosen at random from the data points, but the algorithm weighs the data points according to their squared distance from the closest center already chosen, which has been proven to provide accurate meaningful clusters. Once the centroids are identified, the clusters are built around them based on a distance formula estimated using the number of matches/no matches among vectors, indicating the presence/absence of each functional gene per location. Points with equal distances to more than one centroid were allocated to more than one cluster, assuming each cluster is an open interactive unit, whereby each function can interact with more than one functional cluster. We used the Apriori algorithm [31] to find specific associations (association algorithms find association of the type, e.g., whenever a specific function occurs in A and B, a specific associated function will also occur in A and B; or, if a function occurs in A and B, it will always occur/be absent in C). We also used WEKA to implement the association algorithms. In particular, Apriori seeks simultaneous occurrences of subgroups of attributes, allowing the identification of co-occurrences among subgroups of characteristics (in this case, functions) within specific geographic locations. The levels of significance of the associations thus found are given as confidence levels.

For comparative purposes, the number of genes in a cluster for each functional group was divided by the number of genes in the microarray for that function and presented as the proportional representation of each function within each site, WGCNA module or K++ means cluster. Thus, per each gene functional grouping, the results are shown as the proportion of genes from the category included in the analyses that appear in the cluster/module/site. The full list of functional groupings included in the microarray is shown in Supplementary Table S1.

### 2.5. Comparative Analysis between Function and Taxonomic Distributions

To assess whether the pattern observed in functional distribution was a direct result of phylogenetic distribution, we repeated the WGCNA analysis as above based only on the phylogenetic markers. Subsequently, we used Pearson’s correlation to check whether the geographical distribution of the WGCNA phylogenetic modules followed the distribution of the WGCNA functional modules. A Student’s *t*-test was used to assess whether the correlation was significant at a 5% level of significance.

## 3. Results

### 3.1. Microarray

We analyzed the co-occurrence patterns of over 20,000 genes in a GeoChip microarray, developed from samples from 10 locations from high latitude oceans. The full list of functional groups and functional genes included in the microarray are shown in Supplementary Table S1 (also including rarer functions).

All of the functional genes hybridized with at least one sample in the microarray. The total cover (number of wells in the microarray showing hybridization/total number of spots on a microarray) was 35%, suggesting no noise-hybridization. The expression results we found here are similar to those found from other world locations, giving no reason to doubt that the distribution of these genes across Arctic or Antarctic locations is the result of ecological interactions, rather than a methodological bias.

### 3.2. Diversity Data

**Figure 1:**
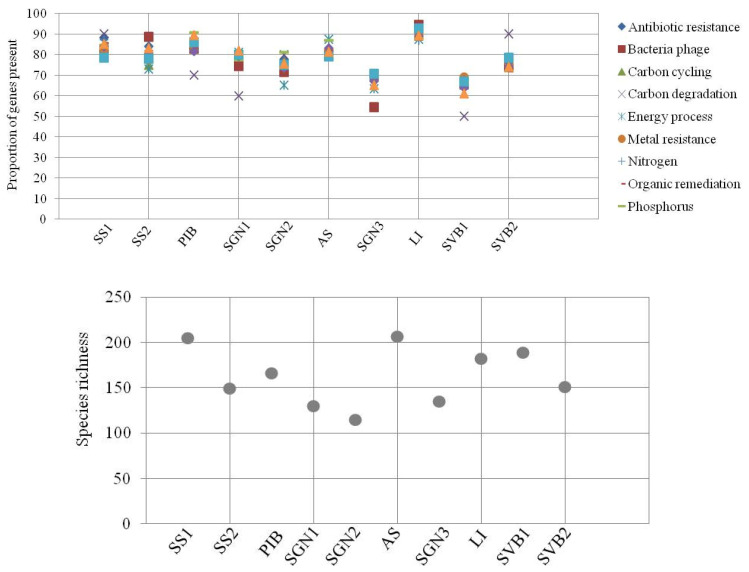
Distribution of functional groups as defined in He et al. 2010 [20] (upper panel) and species richness (lower panel) across sites. Antarctic: SS: South Sandwich; PIB: Pine Island Bay; SGN: South Georgia North; AS: Amundsen Sea; LI: Livingston Island. Arctic: SVB: Svalbard. The data are presented as standardized for the number of functional genes per functional group included in the microarray (as the proportion from all the genes included that is present at each site).

We found significant differences in the distribution of taxonomic species and individual functional genes across sites (pairwise *t*-test with Holm adjustment, *p* < 0.05; Figure 1). In addition, species richness also varied across sites. From all of the genes included in the microarray, the proportion present in each site per function also differed across sites. The number of species detected here was low compared to taxonomy-based studies (as expected given the diversity study was based on a fixed set of markers used for comparative purposes only). This suggests that most of the species in the system are yet to be identified, and consequently, the functional genes here detected come from unidentified or uncultured bacteria species (which is consistent with previous studies).

### 3.3. Functional Networks and Cluster Analysis

**Figure 2:**
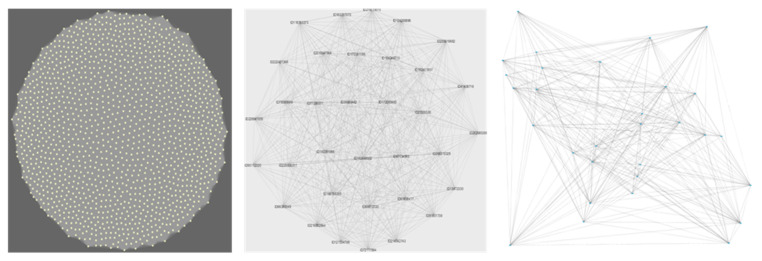
Structural organization of microarray genes by weighted gene co-occurrence network analyses (WGCNA) modules. First panel: Turquoise functional module; Second panel: Royal Blue functional module. Each colour represents a WGCNA module built from functional genes that tend to co-occur (see Methods section for full description); Third panel: Phylo1 module from the WGCNA network built based on only phylogenetic markers. The figure is shown to highlight the underlying structure of a random network.

**Figure 3:**
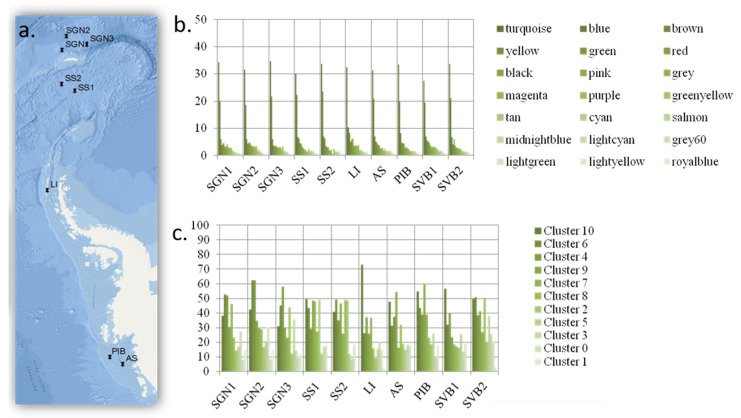
Geographical distribution. (**a**) Geographical location of the study sites in the Antarctic. Antarctic: SGN: South Georgia North; SS: South Sandwich; LI: Livingston Island; AS: Amundsen Sea; PIB: Pine Island Bay. Arctic: SVB: Svalbard. (Note SVB1 and SVB2 are two samples from an Arctic location in Svalbard, not shown in the map). (**b**) distribution of the WGCNA modules. The colour names in the legend of panel a refer to each of the WGCNA modules as defined by the method. (**c**) distribution of the K++ means clusters across Arctic and Antarctic locations. The columns are coloured in a gradient of genetic density per module or cluster (i.e., darker columns represent modules/clusters with more genes, whilst those including less genes are shown in lighter colours). WGCNA module and K++ means cluster distribution did not differ significantly across geographic locations. The data are presented as standardized for the number of genes per functional type included in the microarray. The functions included are listed in Supplementary Table S1.

To understand how functions associate and how microbial networks organize at the functional level, we used weighted gene co-occurrence network analyses (WGCNA), K-means++ cluster analysis and association algorithms. WGCNA was specifically designed to analyze co-occurrence or co-expression patterns based on microarray data [26], and network analyses are particularly useful to represent the level of interconnectivity within interactive units. The WGCNA network analysis based on co-occurrence patterns in situ identified 21 different modules arranged as an Erdős–Rényi (ER) random network (Figure 2). The level of heterogeneity within the modules was low, with most modules presenting an equally high level of connectivity. We tested how the different functional groups distributed across these modules, and we found that the different functional groups distributed homogeneously across geographical locations (Figure 3; Wilcox test; *p* > 0.05). In addition, the distribution of the different WGCNA network modules did not differ significantly (95% CI) between Antarctic and Arctic locations.

The K means++ analysis, including all functional genes, identified 10 clusters whose distribution followed a pattern similar to that found in the WGCNA network analysis (Figure 1) and, in addition, did not differ significantly across sites (Wilcox test, *p* < 0.05).

Our results show that the different functional groups were distributed evenly across geographical locations (Figure 3). In addition, the distribution of the different WGCNA network modules did not differ significantly (95% CI) among all of the Antarctic and Arctic locations, suggesting a homogeneous functional distribution. Furthermore, this WGCNA functional distribution did not follow either species richness, species distribution or the distribution of functional genes alone. This can be seen by comparing the distribution of these parameters between sites shown in Figure 1, and the distribution of WGCNA modules between sites shown in Figure 3. Similarly, analyzing the data using the K++ means clustering algorithm nesting the genes by function, under the assumption that similar functions are subject to similar selective pressures, the distribution of K++ means functional clusters also followed a pattern similar to that found in the WGCNA network analysis (Figure 1), and also did not differ significantly across sites (Wilcox test, *p* < 0.05). Furthermore, association algorithms used to test whether functions associate in any specific manner within the sites did not show any significant association. Only organic remediation and stress appeared to weakly associate, but only at confidence levels below 50%.

### 3.4. Correlation between WGCNA Phylogenetic Modules and WGCNA Functional Modules

**Figure 4:**
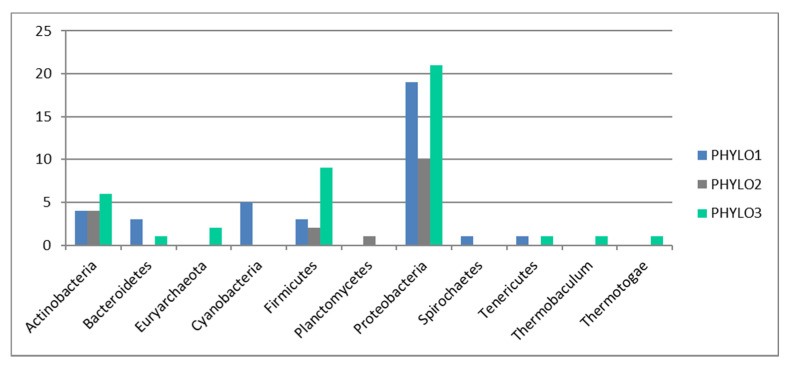
Distribution of taxonomic groups among the three phylogenetic clusters. The data are presented as standardized for the number of genes per bacterial type included in the microarray.

At the phylogenetic level, network analysis using weighted gene co-occurrence analysis (WGCNA) showed only three distinct modules within the genes included in the microarray, suggesting that the functional groupings found also did not correspond to species or taxonomic units. The three phylogenetic modules (PHYLO1, PHYLO2 and PHYLO3) include representatives of all main groups expected from marine samples, including Proteobacteria, Actinobacteria and Bacteriodetes, together with Firmicutes, at different proportions (Figure 4).

**Figure 5:**
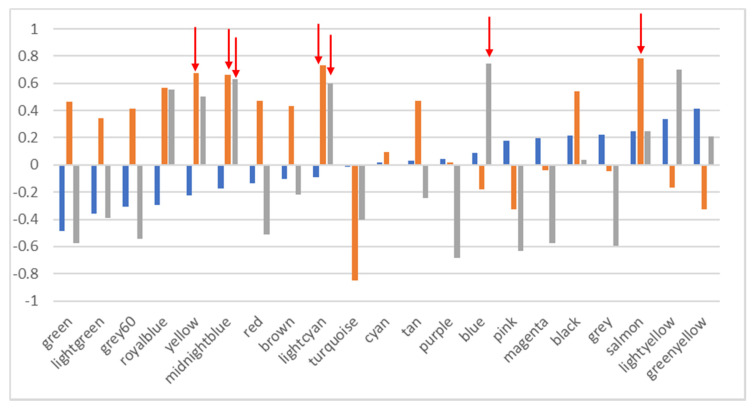
Correlation (y-axis) in the geographical distribution between WGCNA phylogenetic modules and functional WGCNA modules. The three columns represent the correlation coefficient for each of the three phylogenetic modules (Blue: PHYLO1; Orange: PHYLO2; Grey: PHYLO3) with the functional modules on the y-axis. Red arrows highlight the correlations that are significant.

Only five (blue, yellow, salmon, midnight blue and light cyan WGCNA modules) of the 21 functional modules identified by WGCNA presented a strong significant correlation with the distribution of two of the phylogenetic modules identified using the same method (Figure 5). None of the functional WGCNA modules correlated in their geographical distribution with the PHYLO1 WGCNA phylogenetic module. The geographical distribution of the other 16 functional groups did not seem to follow the biogeographical distribution of the samples from the phylogenetic perspective. In other words, the phylogenetic biogeography does not directly correspond to the functional biogeography. Furthermore, functional biogeography cannot be explained by phylogenetic distribution.

### 3.5. Role of Rarer Functions

To understand the role of rarer functions in the overall functional composition, we analyzed how those functions not included in WGCNA modules (not co-occurring with other functions frequently enough to belong to a module, hence rarer functions) were distributed among K++ means clusters and geographic locations. A K++ cluster analysis on these genes showed a tendency to associate in smaller-sized clusters (Supplementary Figure S1), while dominant functions associated together in a larger module and larger clusters.

**Figure 6:**
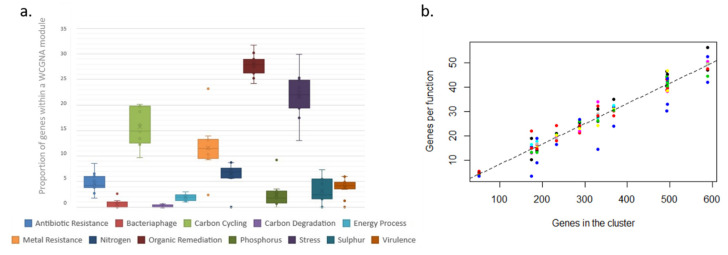
Functional gene distribution within clusters. (**a**) proportion of genes within a WGCNA module. (**b**): linear trend between the number of genes associated to any of the functions and the total number of genes in each KMeans++ cluster; the individual R^2^ values for the linear relations between genes per function and genes in the cluster per cluster vary between 0.71 for virulence genes to 0.99 for stress and carbon cycling genes. The data are presented as standardized for the number of genes per functional type in the microarray.

The total number of functional genes that were included in each WGCNA module differed (this structure was also found for KMeans++ clusters), with some modules including many more genes than others (Figure 6a). The proportion of times a gene must appear to be linked to a specific module or cluster was 8%, meaning the relationship between the number of genes in the cluster and the number of genes per function follows a linear pattern (Figure 6b); thus, smaller clusters require a gene to appear less frequently to get fixed within the cluster than larger clusters, which will require higher gene frequencies.

## 4. Discussion

Initially, we set out to test whether an increase in functional diversity should lead to a proportional increase in the peripheral functions of the expected scale-free network conferring resilience. What we found, however, was a very different picture. The network did not organize as a scale-free network but as a set of random networks (each network represented as a WGCNA module, represented by colours in Figure 3, panel b). In such a structure, each co-occurring module is in itself weak, as every node (gene in this case) has the equivalent effect on the overall module stability (and is proportional to the number of nodes in the network). In other words, every node (gene) removed from the module, e.g., through extinction, influences the overall functioning of the module (which in turns represents an interacting community).

In contrast, the modules and clusters found using two different methods organize in an even manner, based on how frequently or infrequently functional genes linked to each functional group appear in the system. Thus, all functional groups are equally represented within each WCGMA module or K++ means cluster. We find that, in such systems, frequent functions associate together in big clusters, while infrequent ones associate together in smaller clusters. The size of the cluster thus represents the number of genes present per functional group in the cluster. In this case, each functional group includes an equal share of 8% of the genes in the cluster. This way, each module always includes a proportional representation of all the functional groups and there are no functional groups missing on any module or cluster.

Thus, the overall ecosystem is organized in such a way that a different co-occurring module is prepared to take the place of any collapsing module. We do not have an ecological mechanism for this organization, but we hypothesize that an ecosystem organized in such a way would be ready to take over any novel niche it opens in the environment (even if the new niche comes from extinction of the existing community) without the immediate need of genetic adaptation, through reproduction rate alone before evolutionary adaptation for optimal growth kicks in. The mechanism we describe here will not ensure colonization of any environment on its own unless the pre-adapted genetic pool is present but would allow colonizing a new environment as a fully functioning community, potentially speeding up colonization.

Indeed, our results also show a very striking pattern—that functions do not associate following a biological pattern (e.g., we do not find a stress function module or a virulence function, nor do we find functions linked to specific metabolic pathways, e.g., functions linked to carbon degradation do not associate in a particular module). The pattern also does not follow a phylogenetic pattern, as the phylogenetic module grouping (three distinct modules only) does not relate to the 21 functional modules found. A correlation analysis also show that the majority of the patterns found for functional distribution cannot be explained by phylogenetic distribution.

Association algorithms [31] used to test whether there is any specific relationship between functional groups and the K++ means clusters did not provide any significant results either (not even random noise, as would be expected if the functional groups were distributed randomly among the clusters). This further supports the hypothesis that the functional associations (i.e., patterns seen for both WCNGA and K++ means algorithms) we find here are indeed evolutionarily homogeneous among clusters and that this is not the result of a random distribution.

We also found that this homogeneous distribution of functional groups within the WGCNA modules or the K++ means clusters is apparent regardless of the clustering method used. Or, in other words, the results obtained here are not an artifact of the method. Our analysis includes two independent methodologies using different algorithms (WGCNA network analysis and K++ clustering [26,29]) and both return the same proportional association. Furthermore, this association is significant after correcting for the number of functional genes per functional group included in the microarray. The main difference between the results from these two methods (WGCNA module detection and K++ clustering) is that, even if the overall pattern is similar (i.e., even module distribution across sites and more abundant modules dominating the functional landscape), the main module identified by WGCNA includes many more functions than the main cluster in the K++ cluster analysis, and it is also more predominant (i.e., in relation to the predominance of all other modules/clusters) in each site than the main cluster in the cluster analysis. This difference potentially relates to the fact that WGCNA only included those functions occurring in six sites or more (dominant functions), while the K++ means cluster analyses included all genes nested by functional category under the ecological assumption that similar functions are subjected to similar selective pressures (i.e., antibiotic resistance, carbon sequestration, metal resistance, etc.).

This pattern of functional group distribution would result in a resilient system based on a structural redundancy, composed of a series of independent functionally-sound clusters, and therefore for the system to ‘collapse’ or become non-functional all the clusters would need to collapse simultaneously. Furthermore, the pattern found at the functional level does not follow the pattern found for taxonomic diversity. Therefore, the pattern found for functional groups does not represent taxonomic units. This pattern is consistent with the scale-free network patterns found for taxonomic associations, as it would allow for a large number of taxonomic units to be depleted without altering the proportional distribution of functional genes with a minimum level of functional gene redundancy in the system (as each taxonomic unit would need to impair the functionality of all functional clusters before the system became dysfunctional).

If this pattern holds for other polar environments, our analyses would explain why we have not found strong evidence of recent environmental impact on polar marine microbial communities at the functional level (unless all communities analyzed have changed exactly in the same direction and intensity simultaneously, which is unlikely given we are comparing areas changing at different rates [32]). Whilst arguably the temperature threshold might have not been reached for bacterial communities [33], strong changes have been reported for phytoplankton communities from the maritime Antarctic and widespread evidence ecological changes at the taxonomic level is available from both the Arctic and the Antarctic [34,35]. Therefore, even if microbial community composition is changing rapidly in these regions due to climate warming, the change is not yet obvious at the functional level. The pattern we see here is not what we would expect from a genetic erosion scenario either, as all sites include tightly packed complex networks of essential genes and a series of smaller, but well-structured and homogeneously present, clusters of less frequent genes. Genetic erosion will not only be expected to homogenize the landscape, but to reduce functional diversity throughout. The scenario we see here is that of diverse functionally redundant clusters homogeneously distributed, which is a pattern more likely to reflect the very nature of functional gene distribution in microbial communities than a consequence of genetic erosion. Moreover, this pattern holds and is significant despite the large geographical range of the sampling covered, including areas above and below the Antarctic Convergence and an Arctic site. This pattern is also significant and holds despite the temporal variability in the sampling, suggesting again that it is characteristic of the system under study, and not an artefact of seasonal or geographical variation.

Overall, our results show a fairly even functional structure within microbial communities in high latitude oceans based on the presence/absence of specific functional genes and their relative co-occurrence. The sampling sites have repeatedly been acknowledged as some of the fastest warming environments on Earth [17,32]. Such changes, however, do not seem to influence the functional structure of the microbial communities present. Our results also show that 8% of the genes within a cluster are devoted to any particular functional group (Figure 4), suggesting that, in order for a new function to become established within a functional cluster, the proportion of genes involved in the function in relation to the cluster gene pool must surpass a minimum threshold; less frequent functions might not succeed in integrating themselves within functional clusters. This mechanism seems to ensure that microbial networks do not rely on less frequent functions (the extreme of this situation would be ensuring that the ecosystem function does not rely on rare functions). In addition, no function becomes dominant over the others, also reducing the risk of communities relying on a small set of keystone functions. This also raises the question of the importance of rarity for overall ecosystem function. At higher taxonomic levels, rarity seems to be essential to maintain ecosystem resilience in highly diverse ecosystems [36]; however, our results show that a high level of rarity does not necessarily translate into higher levels of redundancy, as there is no link between the distribution of less frequent functions and the distribution of taxonomic diversity. Consequently, less frequent genes do not contribute to the overall ecosystem function in the polar oceans at the ecological time scales considered here, at least for microbial communities.

Here, we have used two different clustering approaches, based on co-occurrence and presence-absence data, to support our conclusions. Whilst the network analysis is based on the strength of the signal in the microarray, the K++ clustering method is based on presence/absence data. These two methods were specifically chosen to reduce the probability of a mathematical bias or a methodological bias based on the strength of the molecular signal. The algorithms used in the network analysis and in the K++ cluster analysis indeed differ considerably, yet the conclusions are significantly similar (a homogeneous distribution of functions among clusters, and clusters among locations). Therefore, even if the level of clustering differs between these two methods (expected as the algorithms differ), the conclusions from these two methods do not.

## 5. Conclusions

Our current understanding of biological networks to date assumes that these networks are arranged in such a way that only a series of key nodes are important and they are in a minority; thus, just by pure probability, for every certain number of genes taken away from the gene pool (e.g., becoming extinct) only one will have an ecological impact. However, we do not find this type of structure in a series of Antarctic and Arctic locations. Instead, we find a structure where the bacterial networks are in themselves weak based on the node structure, because every gene taken out will have an impact. However, the strength of the ecosystem relies on its overall organization, in such a way that a different co-occurring network will take its place once the previous network collapses. Whilst our data does not allow formulating an ecological mechanism for this organization, an ecosystem organized in such a way would be potentially ready to take over any novel niche it opens in the environment (even if the new niche comes from extinction within the existing community) without the need for genetic adaptation, through reproduction rate alone.

In summary, a tight organization of functional groups in self-contained bacterial networks (analyzed here as WGCNA modules/K++ means clusters) might explain functional resilience at the microbial level. Microbes are certainly not immune to ecological change, as their diversity and community dynamics do change following environmental perturbation [37,38]. Indeed, these types of change could speculatively be behind the differences we find in species richness or in the distribution of individual functions. However, our results show a solid functional distribution at the ecosystem level.

## Supplementary Materials

Supplementary materials can be found at https://www.mdpi.com/2076-2607/8/6/951/s1.
