# Supplementary material for: Functional Associations and Resilience in Microbial Communities
Source: Microorganisms. 2020 Jun 24;8(6):951. doi: 10.3390/microorganisms8060951 (PMC7357002; doi:10.3390/microorganisms8060951)
Supplement: Supplementary file 1 [file microorganisms-08-00951-s001.pdf]

Supplementary Materials

Figure S1 Cluster distribution (as defined by K++ Means) of those genes not included in the WGCNA modules by geographic location. The columns represent, in a colour gradient, cluster genetic density (i.e., darker columns represent modules with more genes, whilst those including less genes are shown in lighter colours). Module distribution does not differ significantly across geographic locations. Antarctic: SGN: South Georgia North; SS: South Sandwich; LI: Livingston Island; AS: Amundsen Sea; PIB: Pine Island Bay. Arctic: SVB: Svalbard.

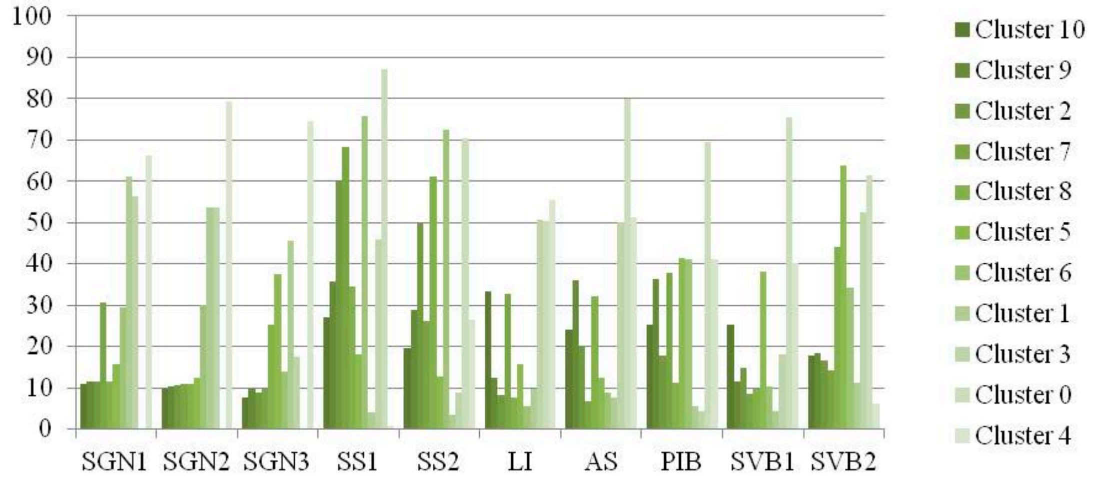

Table S1. List of functional categories included in the microarray in alphabetical order. All genes in the microarray were categorized within one of the following functional categories.

Acetogenesis	Energy	Osmotic stress
Aluminium resistance	Glucose limitation	Oxygen stress
Ammonification	Hydrocarbon remediation	Other
Anammox	Heat shock	Pectin degradation
Antibiotic resistance	Hemicellulose degradation	Pesticides remediation
Antibiotic resistance transporter	Herbicides remediation	Phosphorous
Aromatics remediation	Host recognition	Phosphorous limitation
Arsenic resistance	Lead resistance	Protein stress
Assimilatory nitrogen reduction	Lignin degradation	Phosphorous use
Cadmium resistance	Lysis	Remediation
Carbon degradation	Mercury resistance	Replication
Cellulose degradation	Metal resistance	Sigma factors
Carbon fixation	Methane oxidation	Silver resistance
Chitin degradation	Methane production	Sulphur oxidation
Chorin remediation	Mixed metal resistance	Starch degradation
Chrome resistance	Multi metal resistance	Stress
Cobalt resistance	Nitrogen fixation	Structural phage
Cold shock	Nickel resistance	Sulfite reductase
Colonization factor	Nitrification	Sulphur
Cobalt/Nickel resistance	Nitrogen	Tellurium resistance
Copper resistance	Nitrogen limitation	Type_III_secretion
Denitrification	Oxygen limitation	Virulence
Dissimilatory nitrogen reduction	Organic remediation	Zinc resistance
